# Heart Failure Patients and Implications of Obesity: A Single-Center Retrospective Study

**DOI:** 10.7759/cureus.18140

**Published:** 2021-09-20

**Authors:** Mohsin S Mughal, Ali R Ghani, Sundeep Kumar, Waqas Hanif, Irfan Ahsan, Hafsa Akbar, Sara Aslam, Zain Khakwani, Deana Mikhalkova, Howard Levitt

**Affiliations:** 1 Internal Medicine, Monmouth Medical Center, Long Branch, USA; 2 Cardiovascular Medicine, Saint Louis University, St. Louis, USA; 3 Internal Medicine, University of Central Florida College of Medicine, Orlando, USA; 4 Internal Medicine, Jack D. Weiler Hospital, Montefiore Medical Center, New York, USA; 5 Internal Medicine, Geisinger Medical Center, Danville, USA; 6 Department of Internal Medicine, Abington Memorial Hospital, Abington, USA; 7 Medicine, University Medical and Dental College, Faisalabad, PAK; 8 Interventional Cardiology, Newark Beth Israel Medical Center, Newark, USA; 9 Cardiology, Newark Beth Israel Medical Center, Newark, USA

**Keywords:** heart failure, comorbid obesity, hospital readmission, non-invasive mechanical ventilation, average length of stay

## Abstract

Background and objective

The prevalence of heart failure (HF) is on the rise; currently, it affects around five million people in the United States (US) and the prevalence is expected to rise from 2.42% in 2012 to 2.97% in 2030. HF is a leading cause of hospitalizations and readmissions, accounting for a major economic burden to the US healthcare system. Obesity is a widely accepted risk factor of HF; however, data regarding its independent association with HF mortality and morbidity is heterogeneous. Globally, more than two-thirds of deaths attributable to high body mass index (BMI) are due to cardiovascular diseases (CVD). This study aimed to investigate the potential role of obesity (BMI >30 Kg/m^2^) in HF patients in terms of 30-day readmissions, in-hospital mortality, and the use of noninvasive positive pressure ventilation (NIPPV).

Methods

In this single-center, retrospective study, all adult (age: >18 years) patients who were hospitalized with a primary diagnosis of HF at the Abington Jefferson Hospital from January 2015 to January 2018 were included. Demographic characteristics were collected manually from electronic medical records. Outcomes were 30-day readmission due to HF, all-cause in-hospital mortality, and requirement for NIPPV. Multivariable logistic regression analysis was conducted to investigate the association of obesity with HF outcomes.

Results

A total of 1,000 patients were initially studied, of these 800 patients were included in the final analysis based on the inclusion criteria. Obese patients showed higher odds for 30-day readmissions and the use of NIPPV compared to non-obese patients. There was no significant difference in in-hospital mortality in obese vs. non-obese patients.

Conclusions

Based on our findings, BMI >30 Kg/m^2^ is an independent risk factor for HF readmissions. Additionally, our results highlight the importance of guidelines-directed medical therapy (GDMT) for HF exacerbation, a low threshold for use of NIPPV in obese patients, promotion of lifestyle modifications including weight loss, and early follow-up after discharge to prevent HF readmissions in the obese population.

## Introduction

Heart failure (HF) is a major global health issue, and it results in frequent hospitalizations and high mortality rates. The prevalence of HF has been increasing; currently, it affects around five million people in the United States (US) and the prevalence is expected to rise from 2.42% in 2012 to 2.97% in 2030. In the next decade, the overall prevalence of HF is projected to increase by 23%, with an estimated eight million cases by 2030 [1-2]. HF is the leading cause of hospitalization, with the three-month readmission rate for this group reaching as high as 35%. Although some progress has been made over the past five years in this regard, more than 20% of patients are still readmitted within 30 days and up to 50% by six months [3]. Obesity is a widely accepted risk factor for HF; however, data is heterogeneous regarding its independent association with HF acute severity outcomes [in-patient mortality, readmission, and utilization of noninvasive positive pressure ventilation (NIPPV)]. Globally, more than two-thirds of deaths attributable to high BMI are due to cardiovascular diseases (CVD). Obesity, diabetes, and hypertension (metabolic syndrome) are prevalent in the HF population and contribute to the pathophysiology of congestive HF (CHF) with reduced ejection fraction (HFrEF) and HF with preserved ejection fraction (HFpEF). Morbid obesity [body mass index (BMI) ≥40.0 kg/m²] is a risk factor for patients with CHF and contributes to an increased risk of readmissions among HF patients. HF and obesity are increasingly prevalent and associated with high morbidity, mortality, and healthcare costs worldwide [4-5]. This study aimed to investigate the potential role of obesity in HF patients in terms of 30-day readmissions, in-hospital mortality, and the use of NIPPV.

## Materials and methods

Study design

This was a single-center, retrospective study involving all adult (age: >18 years) patients who were hospitalized with a primary diagnosis of HF at the Abington Jefferson Hospital from January 1, 2015, to January 2018. Inclusion and exclusion criteria are mentioned in Table 1. Demographic characteristics were collected manually from electronic medical records. Continuous variables were presented as the mean, and categorical variables were expressed as percentages. Outcomes were 30-day readmission due to HF, in-hospital mortality, and requirement for NIPPV. Data collection was focused on determining the rate of HF readmissions, in-hospital mortality due to any cause, and the use of noninvasive ventilation in patients over 18 years of age with HF. HF cases were identified based on the illness at the time of presentation. The data collected from medical records included history/physical examination, demographic variables, as well as EKG and echocardiographic findings. Echocardiograms (2D-transthoracic) were performed by trained technicians and examined by a senior cardiologist to document the ejection fraction (EF). Statistical analysis was performed using SPSS Statistics (IBM, Armonk, NY). Categorical data were analyzed using Pearson's chi-squared test, and a p-value of <0.05 was considered statistically significant. Multivariable logistic regression analysis (including age, gender, race, and comorbidities as mentioned in Table 6) was conducted to investigate the association of obesity with HF outcomes. We divided our patients into two major cohorts:

1. Patients with CHF and a BMI <30 Kg/m^2^

2. Patients with CHF and a BMI >30 Kg/m^2^

All adult (age: ≥18 years) patients who were admitted to the Abington Jefferson Hospital from January 2015 to January 2018 with a primary diagnosis of HF [HFpEF, HFrEF, New York Heart Association (NYHA) Class II to IV, the American College of Cardiology (ACC) Stage B to C] with class I-III obesity (30-40 Kg/m^2^) were included. Hospitalized patients with a primary diagnosis of HF on left ventricular assist device (LVAD), outpatient milrinone infusions, and cardiogenic shock patients were excluded from the study. All data were recorded in a de-identified manner to maintain confidentiality. All patients were treated at the same institution with optimal guidelines-directed medical therapy (GDMT).

**Table 1:** Inclusion and exclusion criteria ACC: American College of Cardiology; BMI: body mass index; HFpEF: heart failure with preserved ejection fraction; HFrEF: heart failure with reduced ejection fraction; LVAD: left ventricular assist device; NYHA: New York Heart Association

Inclusion criteria	Exclusion criteria
All patients who were admitted with heart failure exacerbation from 2015 to 2018	LVAD patients
HFpEF	Outpatient milrinone infusions
HFrEF	Cardiogenic shock
Age of 18 years or above	
NYHA Class II to IV	
ACC Stage B to C	
Obese with BMI >30	

## Results

A total of 1,000 patients were initially studied, of which 800 patients were included in the final analysis based on the inclusion criteria. Of these, 70% were older than 65 years, 55% were male, and 45% were female. Hypertension was the most common comorbidity (70%) followed by diabetes mellitus (30%), chronic kidney disease (14.5%), syncope (11.0%), myocardial infarction (10%), cerebrovascular accidents (8.2%), anemia (6%), and peripheral arterial disease (4.3%). Outcomes (30-day readmissions, use of NIPPV, length of hospital stay, in-hospital mortality) are illustrated in Table 2. Obese patients were more likely to receive NIPPV (odds ratio: 3.098, 95% CI: 1.697-5.657, z statistic: 3.683, p=0.0002). Obese patients showed higher odds for 30-day readmission (odds ratio: 1.775, 95% CI: 1.33-2.37, z statistic: 3.87, p=0.0001) compared to non-obese patients with HF (Table 3).

**Table 2:** Outcomes in obese vs. non-obese patients NIPPV: noninvasive positive pressure ventilation

Parameter	Obese patients (n=427)	Non-obese patients (n=373)	Number of patients/total
30-day readmission	60% (n=180)	40% (n=120)	300/800
Use of NIPPV	75% (n=45)	25% (n=15)	60/800
In-hospital mortality	6% (n=6)	4% (n=4)	10/800
Length of hospital stay (mean)	5 days	3 days	NA

**Table 3:** 30-day readmission in obese vs. non-obese patients* *Odds ratio: 1.775, 95% CI: 1.33-2.37 BMI: body mass index

BMI	30-day readmission	No 30-day readmission	Total number of patients	Relative risk
>30	180	229	409	0.44
<30	120	271	391	0.306

There was no significant difference in in-hospital mortality in obese vs. non-obese patients (odds ratio: 1.4404, 95% CI: 0.403-5.14, z statistic: 0.562, p=0.5741). Outpatient follow-up was not examined as a part of the study. Demographic characteristics and comorbidities are illustrated in Tables 4-6.

**Table 4:** Baseline demographics

Demographics	Values
Total patient population	800 (100%)
Age >65 years	70%
Age <65 years	30%
Males	55%
Females	45%

**Table 5:** Percentage of the patient population with secondary medical conditions/risk factors

Comorbidities/risk factors	Percentage of the population
Hypertension	70%
Diabetes mellitus	30%
History of myocardial infarction	10%
History of percutaneous intervention	7.8%
Chronic kidney disease	14.5%
Cerebrovascular accident (CVA)	8.2%
Peripheral arterial disease (PAD)	4.3%
Anemia	6.0%
Syncope	11.0%

**Table 6:** Baseline demography and comorbidities in obese vs. non-obese patients SD: standard deviation

Parameter	Obese cohort	Non-obese cohort
Age in years, mean ±SD	68 ±9	64 ±11
Male, %	54.8%	56.56%
Hypertension, %	70	68
Diabetes mellitus, %	30	27
Prior myocardial infarction, %	10	13
Prior percutaneous intervention, %	8.0	11.0
Chronic kidney disease, %	14.5	16.0
Prior cerebrovascular accident, %	8.2	11
Peripheral arterial disease, %	4.3	3.8
Prior syncope, %	11	10
Anemia, %	6.0	6.3
Smoking, %	34	33
Ejection fraction, mean	30	34

## Discussion

Obesity increases the CVD burden by various physiological mechanisms. It increases insulin resistance, hyperlipidemia, obesity-induced hypertension, development of systemic inflammation, obstructive sleep apnea (OSA), and eventually congestive failure. BMI is a valid and acceptable tool to quantify the severity of obesity; however, it does not account for the content of body or abdominal fat. Regardless, higher BMI has been associated with CVD and poor outcomes [6]. Studies have suggested an inverse relationship between obesity and mortality due to HF. This concept is called the obesity paradox, which refers to lower mortality in obese HF patients than patients who have normal or higher BMI [7]. Our data indicate that obesity is associated with poor outcomes in hospitalized patients with HF. Frequent hospitalization from HF adds to the already strained healthcare provision system in the US. In this study, we noticed an increased 30-day readmission rate in obese patients (BMI >30 kg/m^2^) with a higher incidence of positive pressure ventilation use and increased length of stay (LOS; five days vs. three days) compared to non-obese patients. We noticed no statistically significant difference in terms of in-hospital mortality in our study though other studies have reported a paradoxical relation of obesity with in-hospital mortality [6-7]. Studies have suggested that HF patients with morbid obesity (>40 Kg/m^2^) also have a higher mortality rate [8]. It is reasonable to assume that in-patient mortality in our study is underpowered.

Obesity is a potentially expanding public health issue and contributes to high morbidity and mortality. Obesity or increased BMI is associated with increased risk and severity of HF [9]. Increased BMI is considered a risk factor for other CVD including hypertension, diabetes, and hyperlipidemia, and all of these increase the risk of myocardial infarction, which in turn augments the risk of HF [10-11]. Elevated BMI is associated with left ventricular remodeling, and the activation of the renin-angiotensin-aldosterone axis increases sympathetic drive and inflammation [12-15]. Studies have indicated that NIPPV methods, continuous positive airway pressure (CPAP), and bilevel positive airway pressure (BiPAP) can effectively treat CHF exacerbations. This supports our finding that obese patients were more likely to receive NIPPV compared to non-obese patients. A significant decline in HF hospitalization is observed in patients after bariatric surgery [16-17]. A grade associated between increasing weight loss and HF reduction has been observed secondary to improvement in diabetes, hypertriglyceridemia, and hypertension. We suggest the continuation of GDMT for HF exacerbation, a low threshold for the use of NIPPV in obese patients, promotion of lifestyle modifications including weight loss, and early follow-up after discharge to prevent HF readmissions in the obese population.

Our study has several limitations, including the fact that it was a single-center study, its retrospective study design, and lack of outpatient follow-up. It is plausible that the stratification of HF into HFrEF and HFpEF may produce different results. Future studies are warranted to further the investigation into obesity by stratifying the etiology of HF and its implications in the HF population.

## Conclusions

Our study suggests that BMI >30 Kg/m^2^ is an independent risk factor for HF readmissions. Furthermore, this data underscores the importance of GDMT for HF exacerbation, a low threshold for the use of NIPPV in obese patients, promotion of lifestyle modifications including weight loss, and early follow-up after discharge to prevent HF readmissions in the obese population.
